# Pre and post-operative self-care management among women undergoing hysterectomy

**DOI:** 10.6026/97320630019721

**Published:** 2023-06-30

**Authors:** Amita Shilpa Gottlieb, Sivasubramanian N, Rajeshbhai Patel Chilka, Mahalakshmi B, Bharatbhai Patel Daminiben, Gopal R

**Affiliations:** 1Graphic Era College of Nursing, Graphic Era Deemed to be University, Dehradun, Uttrakhand -248002, India; Nootan College of Nursing, Sankalchand Patel University, Visnagar, Gujarat -384315, India

**Keywords:** Pre-operative and post-operative, self-care management, hysterectomy

## Abstract

Surgery is a major part in the field of Gynecology. Hysterectomy is the removal of the uterus which is leading non-obstetric surgery worldwide, after caesarean sections. The objective of the current study was to determine how well women undergoing hysterectomy surgery managed their pre-operative and post-operative self-care. A quasi experimental single group pre-test and posttest design was used for this inquiry. Pre - and post-operative self-care management questionnaires were used to collect data from 60 women. Utilizing descriptive and inferential statistics like mean, standard deviation, and chi-square test, data were gathered and examined. Data analysis revealed, the mean post-test knowledge score (23.81) was greater than the mean pre-test knowledge score (10.33). The average disparity was (13.48) with the estimated "t" value (16.003), which was more than the table value (1.67) at 0.05 level of significance, showing that the self-care management education program was successful in increasing women's understanding on hysterectomy. The study concluded that pre- and post-operative self-care management education program had significantly improved health status among women underwent hysterectomy.

## Background:

Hysterectomy is the removal of the uterus which is the leading non-obstetric surgery worldwide, after caesarean sections [1]. Charles Clay carried out the first hysterectomy in November 1843. It was done to get rid of a big myomatous uterus. The World Health Organization estimates that 1,540,000 women worldwide had hysterectomy in 2016, making this the most prevalent major surgery for women that aren't related to pregnancy. By the time they reach the age of 65 years, between 37-39% of women have had hysterectomy, with this procedure being most frequently performed on women between the ages of 40 and 45 [2]. In India, the prevalence of hysterectomy surgery was 3.2%, with Andhra Pradesh having the highest rate (8.9%) and Assam having the lowest rate (0.9%). In 126 districts, the prevalence of hysterectomy was between 3% and 5%, between 5% and 7% in 47 districts, and over 7% in 26 districts. There were substantial neighbourhood associations in 202 districts [3]. Different types of hysterectomy procedures are carried out. 1) A total hysterectomy involves the removal of the womb and cervix. 2) A subtotal hysterectomy involves removing the womb's main body while leaving the cervix in place. 3) The womb, cervix, fallopian tubes, and ovaries are removed during a total hysterectomy with bilateral salpingo-oophorectomy. 4) In a radical hysterectomy, the womb and all surrounding tissues-including the ovaries, fallopian tubes, a portion of the vagina, and fatty tissue-are removed. There are three different techniques for doing a hysterectomy surgery: laparoscopic, vaginal, and abdominal [4].Self-care before and after surgery is crucial for a healthy life. Self-care measures before the surgery, by enhancing the general health include losing weight, quitting smoking and drinking, and taking multivitamins etc. The length of recovery following surgery will vary based on the procedure, the patient's age, and overall health. The self-care management involves getting enough rest, avoiding heavy lifting, not driving; avoid getting constipated, using painkillers as directed, and refraining from sexual activity for up to 8 weeks after surgery [5]. Infectious reasons, venous thromboembolic illness, harm to the genitourinary and gastrointestinal tracts, bleeding, nerve injury, and vaginal cuff dehiscence can be categorized as the most frequent hysterectomy consequences. After an abdominal hysterectomy, pelvic organ prolapse, pelvic organ fistula, urinary incontinence, and intestinal ileus are additional potential consequences. When compared to other minimally invasive hysterectomy techniques like laparoscopy, abdominal hysterectomy has been found to have a higher risk of problems overall and a higher likelihood of postoperative complications within 30 days of operation. [6] The use of a radical hysterectomy procedure that spares the nerves results in a considerable decrease in postoperative bladder morbidity. A well-known procedure, radical vaginal hysterectomy with laparoscopic lymph node dissection, provides great cure rates without abdominal entrance and lower postoperative febrile and gastrointestinal morbidity. Total laparoscopic radical hysterectomy is a less invasive option to a typical abdominal radical hysterectomy procedure that produces a similar safety profile with a significant decrease in blood loss and hospital stay [7]. Patient should do shower, enema or vaginal douche before the surgery. The patient will be given an intravenous antibiotic medicine just prior to surgery to reduce the chance of infection following the treatment. After the abdominal hysterectomy typically necessitates a one or two-days hospital stays, though this can vary. To control vaginal bleeding and discharge, the patient need to wear sanitary pads. After a hysterectomy, bloody vaginal leakage is typical for a few days to weeks. However, the surgeon should know if there is continuous bleeding or bleeding that is as heavy as a menstrual period [8].

Hence the researcher imparted a self-care management strategy in the pre and post-operative period to enhance the wellbeing of women who had undergone hysterectomy surgery.

## Methodology:

The purpose of the current study was to evaluate the effectiveness of teaching program aimed at hysterectomy patients' pre- and post-operative self-care management. A quasi experimental research design was used to complete the study. A quasi experimental single group pre-test and posttest design was used for this inquiry. Pre and post-operative self-are management questionnaires were used. The sample size was 60 women, who were in the phase of preoperative and postoperative care. Data were gathered and examined using descriptive and inferential statistics like mean, standard deviation, frequency, percentage, t-test and chi-square test. The knowledge rating scale scores were ranged from 0 to 30. Each item was answered on a five point scale. Items were scored and graded as,

0 - 10 = Inadequate

11 - 20 = Moderate Adequate

21 - 30 = Adequate

## Results:

According to the demographic distribution of the sample, around 4 (6.66%) women belongs 30-39 years, 26 (43.33%) in 40-49 age group, and 30 (50%) of the women above the age group 50 years. With regard to Marital status, 35(58.33%) of women were married, 5(8.33%) of women were divorced or separated and 20(33.33%) of women were widow. As per the Educational status, 23(38.33%) of women had no formal education, 15(25%) of women had lower primary education, 17(28.33%) of women had high school education and 5(8.33%) women had graduation and above education. With regard to number of children, 5(8.33%) of women having only one child, 30(50%) of women having two children, 25(41.66%) of women were having three and above children. According to the previous history of surgery, 22(36.66%) of women had previous history of surgery and 38(63.33%) of women had no any previous history of surgery. With regard to the occupation of women, 36(60%) of women were housewives, 12(20%) of women were laborer, 8(13.33%) of women were into business and 4(6.66%) of women had government job. According to the type of family, 23(38.33%) of women belongs to nuclear family, 37(61.66%) of women belongs to joint family and no one women from extended family among the study population.

**Figure 1:**
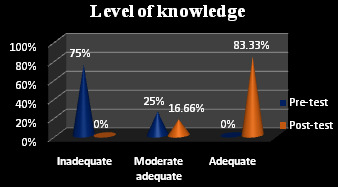
Pre-test and post-test knowledge of women regarding hysterectomy

Figure 1 demonstrates that the pre-test sample, 75% had insufficient awareness of hysterectomy. In the post-test, there was noticeable improvement in the sample knowledge, 16.66% of average knowledge and 83.33% were gained good knowledge. Table 1 shows that after the intervention, the mean score of 23.81 was greater than the mean score of 10.33, before the intervention. The average disparity was (13.48). The estimated "t" value (16.003) was larger than the t - test value 1.67, at 0.05 levels, showing the effectiveness of self-care management in increasing women's knowledge of hysterectomy.

## Discussion:

The current study findings show that there is a significant difference between women's knowledge score on pre- and post-operative self-care management following hysterectomy. Another study was supported by Graw found that, before surgery, 58% of women received information from doctors and 42% of women had prior knowledge of hysterectomy. The majority of women-around 66%- were said; they desired to know more about the effects of hysterectomy [2].Another study result was conducted on the assessment of consciousness, perceptions, and experiences in the hysterectomy decision-making process. The result shows that significant correlations were found between hysterectomy and education, urban location, and women's socioeconomic status. This study found that pre-operative education might help to minimize the post-operative symptoms of women had hysterectomy [9].Another study was carried out in south India, and the subjects found the self-instructional module on hysterectomy to be more helpful in identifying potential post-operative issues like pain in the abdomen, pain at the surgical site, headache, abdominal discomfort, insomnia, fatigue, and anxiety and taking preventive measures. SIM worked well because users could read and get answers to their questions at home [10]. A hysterectomy education programme enhances both physical and sexual well-being after surgery, although the abdominal route had a greater impact on sexual well-being than the vaginal method [11]. According to a different study, significant improvements in all three areas of health status-symptoms, psychological function, and quality of life-were observed following hysterectomy and maintained or got better over the course of the subsequent two years [12]. Another study found that, to reduce problems, adequate training and monitoring are essential prior to beginning a total laparoscopic hysterectomy or a vaginal hysterectomy. It is also crucial to discuss the methods employed by various surgeons, the outcomes, and the complications related to this procedure, especially in the case of total laparoscopic hysterectomy [13]. Another study had established the need for ongoing self-care monitoring to assess the influence on women's health and determine whether the trends have persisted. Future hysterectomy surveillance may require knowledge of both inpatient and outpatient procedures [14]. The most frequent causes of hysterectomy include leiomyomas, abnormal uterine haemorrhage, and pelvic relaxing. When a woman has a hysterectomy, one-fourth to one-half of them have morbidity, the most frequent of which are fever and bleeding. The hysterectomy will still play a significant role in women's healthcare. Each hysterectomy done should be both acceptable and safe thanks to good training techniques and ongoing self-care activities [15].

## Conclusion:

Hysterectomy may be the most economical course of action, but due to its invasiveness and higher risk of complications, it is only seen as a last resort by gynecological professionals and patients who are influenced by other factors like accessibility to care, degree of invasiveness, long-term effects, and patient autonomy. While many gynecological disorders, other than cancer, can be successfully treated using alternative techniques, hysterectomy is advised in cases where other techniques fail. The current study found that teaching programmes on pre and post-operative self-care management significantly improved health status among women who had hysterectomy, and there was a significant difference between pre-test knowledge scores and post-test knowledge scores.

## Figures and Tables

**Table 1:** Mean, Standard Deviation, Mean difference and't' value of knowledge scores before and after education on self-care management

**Parameter**	**Mean**	**Standard deviation**	**Mean difference**	**'t'value**	**Table 't' value**	**Level of Significance 0.05**
Pre-test	10.33
23.81	5.16
Post-test	3.49	13.48	16.003	1.671	S
